# Alleviating iatrogenic effects of paclitaxel via anti-inflammatory treatment

**DOI:** 10.21203/rs.3.rs-2487922/v1

**Published:** 2023-01-30

**Authors:** Mengwei Zhang, Saran Lotfollahzadeh, Nagla Elzinad, Xiaosheng Yang, Murad Elsadawi, Adam Gower, Mostafa Belghasem, Tarek Shazly, Vijaya B. Kolachalama, Vipul Chitalia

**Affiliations:** Boston University; Boston University; Boston University; Boston University; Boston University; Boston University; Boston University; University of South Carolina; Boston University; Boston University

**Keywords:** Peripheral artery disease, drug coated balloon, paclitaxel, dexamethasone, inflammation

## Abstract

**Background:**

Paclitaxel is touted as an essential medicine due to its extensive use as a chemotherapeutic for various cancers and an antiproliferative agent for restenosis. Due to recent concerns related to long-term mortality, paclitaxel (PTX)-based endovascular therapy is now surrounded by controversies.

**Objective:**

Examine the inflammatory mediators driven by the systemic administration of PTX and explore the means to suppress these effects.

**Methods:**

RNAseq analysis, cell and mouse models.

**Results:**

RNAseq analysis of primary human endothelial cells (ECs) treated with PTX demonstrated transcriptional perturbations of a set of pro-inflammatory mediators, including monocyte chemoattractant protein-1 (MCP-1) and CD137, which were validated in EC lysates. These perturbations were abrogated with dexamethasone, a prototypic anti-inflammatory compound. The media of ECs pre-treated with PTX showed a significant increase in MCP-1 levels, which were reverted to baseline levels with DEX treatment. A group of mice harvested at different time points after PTX injection were analyzed for immediate and delayed effects of PTX. A 3-fold increase in MCP-1 was noted in blood and aortic ECs after 12 hours of PTX treatment. Similar changes in CD137 and downstream mediators such as tissue factor, VCAM-1 and E-selectin were noted in aortic ECs.

**Conclusions:**

Our study shows that systemic PTX exposure upregulates atherothrombotic markers, and co-delivery of DEX can subdue the untoward toxic effects. Long-term studies are needed to probe the mechanisms driving systemic complications of PTX-based therapies and evaluate the clinical potential of DEX to mitigate risk.

## 1. Introduction

Peripheral artery disease (PAD) results from atherothrombotic occlusion of the large and medium-sized arteries in the lower limb and is the third leading cause of atherosclerotic morbidity after coronary artery disease (CAD) and cerebrovascular disease^1–3^. The prevalence of PAD in the United States for people over 40 years of age is 8.5 million, with risk increasing substantially with age. Percutaneous transluminal angioplasty (PTA) constitutes the mainstay of therapy for endovascular revascularization^4^.

Drug-coated balloon (DCB) endovascular therapy is an attractive approach to inhibit restenosis within the femoropopliteal and coronary vasculature. Paclitaxel (PTX) is a potent inhibitor of microtubular assembly and cell division^4^, and the therapeutic agent present on all clinically approved DCBs. A recent meta-analysis of randomized trials demonstrated that PTX-treated patients experienced an increased late mortality rate^5^. This result prompted the United States Food and Drug Administration (FDA) to issue a warning that ultimately led to a marked reduction of the clinical use of PTX-based stents and balloons. While there is no known causal mechanism connecting PTX-based therapy with mortality, the FDA’s response to limiting the use of these devices has left a void within the interventional community for treating patients, most notably those with PAD.

Since the FDA’s warning was related to evidence of systemic toxicity in PTX-based devices, we sought to evaluate the downstream systemic effects that are triggered after PTX exposure in cell culture and mouse models. We hypothesized that increase in PTX concentration due to systemic exposure triggers a cascade of biochemical events that can alter the signaling of cytokine and pro-inflammatory pathways, leading to iatrogenesis. To minimize these downstream events, we used the clinically approved anti-inflammatory agent dexamethasone in conjunction with PTX delivery and evaluated the potential to abrogate systemic toxicity. Our study proposes compensatory strategies to minimize untoward PTX-induced effects, which is needed to rejuvenate interest in PTX-coated devices to treat obstructive arterial disease.

## 2. Methods

### Study design

**Figure 1:**
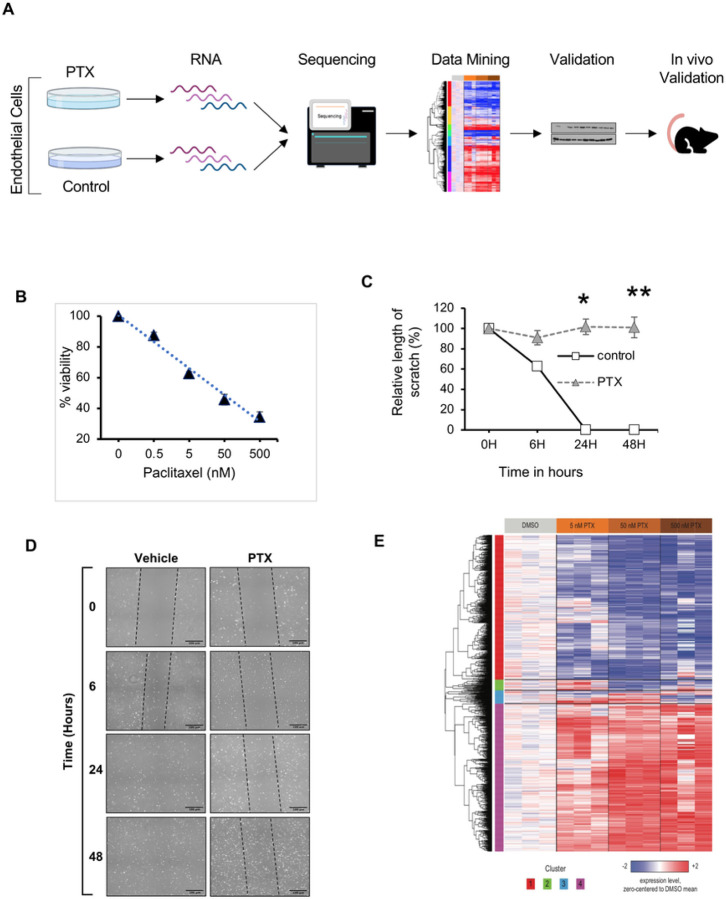
Paclitaxel negatively inhibits EC cell function and induces genetic perturbation. **(A)**. Schematic of the overall experimental strategy to evaluate PTX-induced adverse effects. (**B**). Cell viability assay. Primary human umbilical vein endothelial cells (ECs) were treated with DMSO or PTX (0.5, 5, 50 and 500 nM) for 18 hours. Resorufin fluorescence was measured at 590 nm after 4 hours. Three independent experiments were performed; values and error bars indicate mean ± SEM. Linear regression was used to compute R^2^ = 0.45 and P < 0.001. (**C**). Quantification of EC migration assay. Scratch closure is expressed as relative length across the scar. PTX-treated samples were compared with controls at each time point using Student’s t test. At 6 hours *P = 0.002, 24 hours **P < 0.001, ^##^48 hours *P* < 0.001. (D). Representative bright-field images of migration assay using ECs in 6-well plates. Cells were treated with vehicle or 5 nM PTX for 18 hours. A scratch was induced on the monolayer of cells and images were taken at the indicated time. Black dotted lines highlight the scratch outline. Scale bar = 100 μm. (E). PTX-dependent changes in gene expression. RNA sequencing was performed using ECs treated with DMSO control or PTX (5, 50, or 500 nM) for 24 hours. The heatmap shows the expression of 1,766 genes (rows) with significant differential regulation (ANOVA FDR *q* < 0.25) with respect to PTX concentration across all samples (columns). Rows are ordered by unsupervised hierarchical clustering, and the number of each cluster is indicated below the heatmap. Expression values for each gene were zero-centered relative to the DMSO control group, and then trimmed to the range −2 to +2, with blue, white, and red indicating zero-centered values of ≤ −2, 0, and ≥ 2, respectively.

All animal experiments were conducted after the IACUC approval from Boston University and Boston University Medical Campus. All procedures conformed to the guidelines from Directive 2010/63/EU of the European Parliament on the protection of animals used for scientific purposes or the NIH Guide for the Care and Use of Laboratory Animals. Figure 1A shows the overview of the study including the cell culture models, viability, migration and immunosorbent assays, RNA sequencing and mouse models. The study is reported in accordance with ARRIVE guidelines.

### Cell culture

Pooled human umbilical vascular endothelial cells (ECs) (Cat No. C2519AS) were obtained from Lonza and grown in EGM-2 media made from EBM-2 media supplemented with an EGM-2 BulletKit (Cat No. CC-3162; Lonza). Cells were incubated in a 37°C humidified incubator with 5% CO_2_. ECs were grown in gelatin-coated plates. The culture plates were coated with 2% gelatin (Cat No. G1890; Sigma) and air dried for at least 1 hour before use.

### Drugs

Paclitaxel (PTX) (Cat No. P9600; LC Laboratories) and dexamethasone (DEX) (Cat No. 11015; Cayman Chemical Company) were dissolved in DMSO (Cat No. D8418–250ML; Sigma).

### Cell viability assay

ECs seeded in 96-well plates underwent survival assay using alamarBlue solution (Cat No. BUF012B; Bio-Rad) per the manufacturer’s instructions.

### RNA extraction

ECs were seeded in p60 plates, treated with DMSO and PTX at ~ 60% confluence for 24h, and harvested for RNA extraction following the RNeasy Mini Kit protocol (Cat No. 74104; Qiagen). RNA was eluted with 40 μL of RNase-free water. RNA concentration was measured with a spectrophotometer (BioDrop).

### Antibodies

Primary antibodies used were CKS2 (Cat No. ab155078; Abcam), BMF (Cat No. 50542S; Cell Signaling), CD137 (34594S; Cell Signaling), MCP-1 (Cat No. 81559S; Cell Signaling), HSP90 (Cat No. 8165S; Cell Signaling). Proteins were visualized with chemiluminescent substrate (Cat No. 34577; Thermo Scientific) and a developing machine (Evolve).

### Migration assay and imaging

EC cells were grown in 6-well plates until 60% confluency and treated with drugs for 18h. A single scratch with a P1000 pipette tip was induced on the monolayer. Media was aspirated and 2 ml of new drug-treated media was added after scratch. Images were taken at baseline, immediately after scratch, and at 6 hours, 24 hours and 48 hours post-scratch.

### Image processing

All images were taken with a Nikon Eclipse Ti inverted microscope with bright field camera at the Boston University School of Medicine (BUSM) Imaging Core. NIS-Elements software was the platform used for controlling the microscope and documenting photos. Six fields of 10X images were taken randomly with phase 1 lenses to create a wide-field image. X, Y and Z coordinates of the scratch in each well were saved onto the microscopy software so that each time point would have the image at the same location to reduce variability. Images were exported as full-resolution tagged image le format (TIFF) and quantified with ImageJ software. The “straight line” tool was used to make a random horizontal straight line across the scratch to measure the distance of the gap in three different locations of the same sample. Each sample was repeated in triplicate. The distance of the gap was taken at a random location for the same well at different time points. The straight line was then added to the “ROI manager” and values were measured in pixels.

### Animal model

A group of female C57BL/6, 8–10 week old mice were obtained from Jackson Laboratories and housed by Boston University Medical Center with approval from the Institutional Animal Care and Use Committee (IACUC AN-1549). DEX and PTX were dissolved in DMSO and further diluted in normal saline for intraperitoneal (IP) injections at 5 mg/kg. Isoflurane inhalation was used as an anesthetic agent. Specifically, we used 2% inhalation for induction and 1% isoflurane inhalation for maintenance using a nose cone. Euthanasia was induced via CO2 inhalation in a pre-charged chamber followed by secondary euthanasia by thoracotomy. These methods are consistent with the recommendations of the Panel on Euthanasia of the American Veterinary Medical Association. Serum, heart, kidney, liver, and aorta were collected at either 20 minutes or 12 hours after IP injection. Serum underwent ELISA and LC/MS studies to examine various inflammatory cytokine levels and serum PTX levels. Mouse aortas were subjected to immunofluorescence staining and protein analysis.

### Cytokine ELISA panel

ECs plated in 6-well plates were treated with vehicle, PTX, DEX, or PTX + DEX. 300 μl of media was collected and sent to Quansys Biosciences (Utah, USA) to perform the Human Cytokine Release Syndrome (16-plex) ELISA panel. A total of 150 μl of mouse serum was collected and sent to Quansys Biosciences to perform the Mouse Cytokine (6-plex) ELISA panel.

Other methods, including immunoblotting, antibodies, immunohistochemistry, immunofluorescence, and liquid chromatography/mass spectrometry are included in the supplementary information.

### RNA sequencing and analysis

STAR (version 2.6.0c) was used to align sequence reads to human genome build hg38. FASTQ quality was assessed using FastQC (version 0.11.7), and alignment quality was assessed using RSeQC (version 3.0.0). Ensembl-Gene-level counts for non-mitochondrial genes were generated using featureCounts (Subread package, version 1.6.2) and Ensembl annotation build 100 (uniquely aligned proper pairs, same strand). Differential expression was assessed using the likelihood ratio test implemented in the DESeq2 R package (version 1.23.10) to perform a one-way analysis of variance (ANOVA) with respect to PTX concentration. Benjamini-Hochberg False Discovery Rate (FDR) correction was applied to obtain FDR-corrected *p* values (i.e., FDR *q* values). The web-based tool Enrichr (https://maayanlab.cloud/Enrichr/) was used to identify pathways and processes included in the MSigDB Hallmark 2020 collection that are significantly overrepresented within clusters of genes.

### Statistical analyses

Parameters were expressed as mean, median, standard deviation, and standard error of the mean (SEM). A comparison of groups was performed using independent Students *t* tests where a *P* value less than 0.05 was considered significant. In selected cases, adjustments were performed for multiple comparisons using Bonferroni correction. All *P* values less than 0.05 were deemed significant as statistical analyses were performed using Excel and Prism software.

## 3. Results

### Paclitaxel compromises fundamental endothelial cell function and induces pro-inflammatory genes

We first examined the effect of PTX in primary human umbilical vein endothelial cells (ECs). ECs were treated with PTX for 18 hours and DMSO-treated cells served as controls. PTX significantly reduced cell viability in a dose-dependent manner, reaching half-maximal inhibitory concentration (IC_50_) at 50 nM (Fig. 1B). The effect of PTX on EC migration was examined using a scratch assay, where a monolayer of ECs was injured following treatment with PTX for 18 hours (Figs. 1C & 1D). The scar completely closed in the control group while the scar in the PTX-treated ECs remained unaltered over 48 hours, suggesting that PTX significantly suppresses EC viability and migration.

RNA sequencing was then performed using ECs treated with PTX over a range of concentrations centered on its IC_50_ (5, 50, and 500 nM). A one-way analysis of variance (ANOVA) was used to identify genes that are differentially regulated across PTX concentrations, and the 1,766 genes with the greatest significance (FDR *q* < 0.25) were divided into four groups by hierarchical clustering (Fig. 1E), including two large clusters of genes that were uniformly up- or down-regulated across all three concentrations of PTX. The tool Enrichr (see Methods) was then used to identify pathways and processes that are significantly overrepresented (adjusted *p* < 0.05) within each of these two clusters (Table 1). Cluster 1 (down-regulated by PTX) was enriched in genes associated with DNA repair, whereas cluster 4 (up-regulated by PTX) was enriched in genes associated with mitotic spindle formation (in accordance with the effect of PTX on microtubule assembly) as well as many inflammatory processes, including TNF-α signaling via NFkB and IL-2 and IL-6 signaling. We focused on several genes represented in these inflammatory gene sets, including cyclin-dependent kinases regulatory subunit 2 (CKS2), CD137 (TNFRSF9), and monocyte chemoattractant protein 1 (MCP-1/CCL2), due to their association with vascular diseases. CKS2 interacts with cyclin-dependent kinases to regulate the cell cycle and is associated with atherosclerosis^6^. CD137 mediates adhesion molecules on ECs and mediates EC dysfunction and pro-inflammatory cytokine response^7,8^. MCP-1 is a well-established pro-inflammatory cytokine associated with atherothrombotic diseases^41,42^. All these observations raised the possibility of inflammation as a contributory factor to the effects of PTX and formed the rationale for using dexamethasone (DEX), a prototypic anti-inflammatory agent, to abrogate these effects.

### Dexamethasone abrogates transcriptional perturbations modulated by PTX in ECs

**Figure 2:**
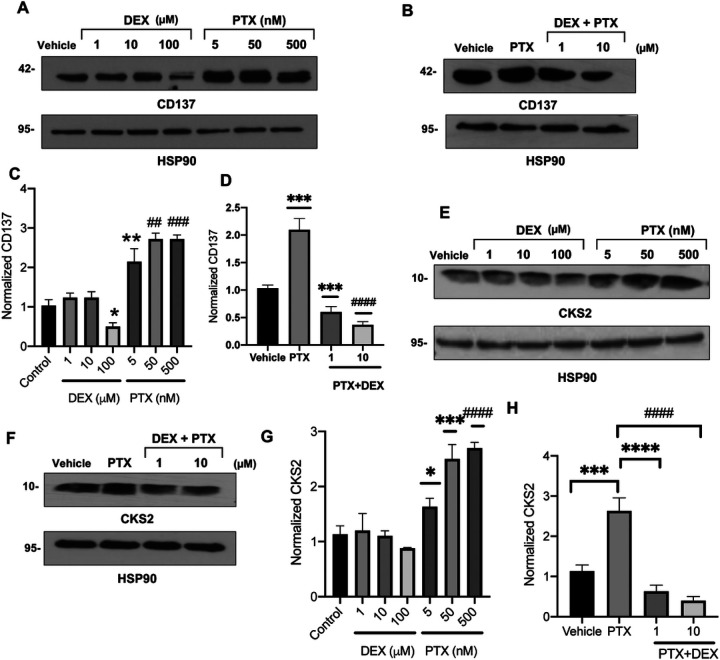
Regulation of CD137 and CKS2 protein expression in ECs by PTX and DEX. (**A**). Immunoblotting of CD137 in ECs. Lysates of ECs treated with vehicle, DEX, or PTX for 24 hours were probed for CD137. HSP90 was used as loading control. Representative images of three independent experiments are shown. (**B**). Immunoblotting of CD137 in ECs. Lysates of ECs treated with vehicle, 5 nM PTX, or 5 nM PTX + 1 μM or 10 μM DEX for 24 hours were probed for CD137. HSP90 was used as loading control. Representative images of three independent experiments are shown. (**C**). Normalized CD137 expression. Bars indicate mean of three independent experiments ± SEM. Student’s *t* test was performed between each treatment and vehicle control. *P = 0.042, **P = 0.004, ^##^*P* = 0.002, ^###^*P* < 0.001. (D). Normalized CD137 expression. Bars indicate mean of three independent experiments ± SEM. Student’s *t* test was performed between each treatment and vehicle control.***P = 0.001, ***P = 0.003, ^####^*P* < 0.001. (**E**). Immunoblotting of CKS2 in ECs. Lysates of ECs treated with vehicle, DEX, or PTX for 24 hours were probed for CKS2. HSP90 was used as loading control. Representative images of three independent experiments are shown. (**F**). Immunoblotting of CKS2 in ECs. Lysates of ECs treated with vehicle, 5 nM PTX, or 5 nM PTX + 1 μM or 10 μM DEX for 24 hours were probed for CKS2. HSP90 was used as loading control. Representative images of three independent experiments are shown. (**G**). Normalized CKS2 expression. Bars indicate mean of three independent experiments ± SEM. Student’s *t* test was performed between each treatment and vehicle control. *P = 0.036, ***P = 0.019, ^###^*P* < 0.001. (**H**). Normalized CKS2 expression. Bars indicate mean of three independent experiments ± SEM. Student’s *t* tests were performed between pairs of treatments. ***P = 0.036, ****P < 0.001, ^####^*P* < 0.001.

Next, we validated specific transcriptional perturbations at the protein level by treating ECs with titrated concentrations of PTX or DEX, with the hypothesis that DEX will revert changes in expression induced by PTX. Treatment with concentrations of PTX as low as 5 nM increased CD137 expression by 2.5-fold (P = 0.004) (Fig. 2A and 2C). DEX treatment alone had a minimal effect on CD137 levels, except for a marginal downregulation of CD137 at 100 uM DEX (P = 0.042); however, co-treatment with DEX prevented the induction of CD137 expression by PTX (Fig. 2B and 2D). Similarly, PTX upregulated CKS2 expression in ECs in a dose-dependent manner, which was prevented by co-treatment with DEX (Fig. 2E–2H). Immunoblot analysis also confirmed the observation by RNAseq that BMF expression is down-regulated by PTX (**Supplementary Fig. 1A-1D**), and showed that it was upregulated in a dose-dependent manner by DEX.

### Dexamethasone prevents the induction of MCP-1 by PTX in vitro

**Figure 3:**
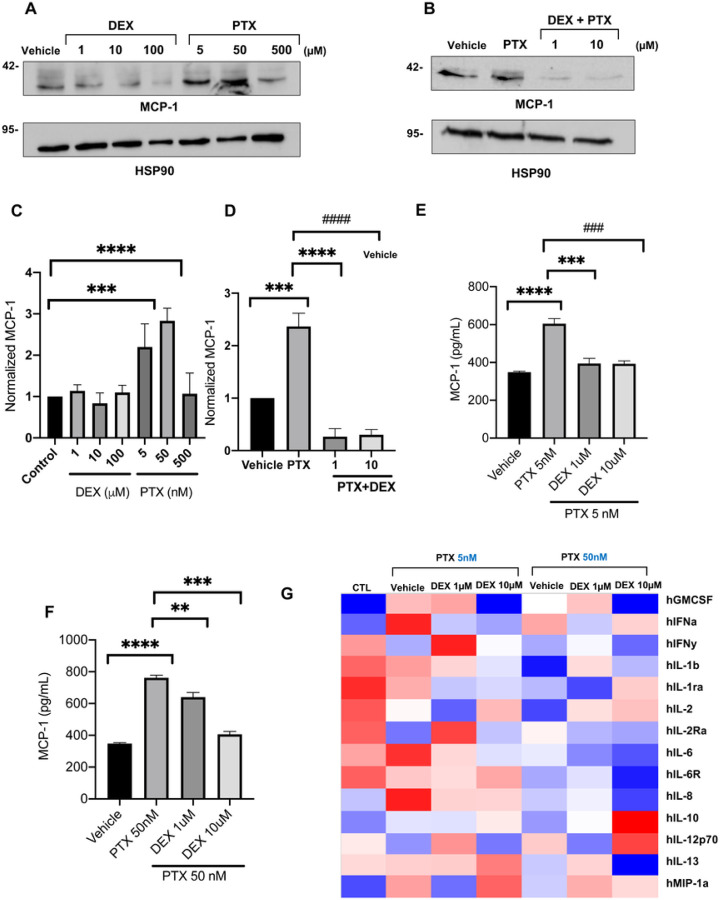
Regulation of MCP-1 and secreted inflammatory cytokines in ECs by PTX and DEX. (**A**). Immunoblotting of MCP-1 in ECs. Lysates of ECs treated with vehicle, DEX, or PTX for 24 hours were probed for MCP-1. HSP90 was used as loading control. Representative images of three independent experiments are shown. (**B**). Immunoblotting of MCP-1 in ECs. Lysates of ECs treated with vehicle, 5 nM PTX, or 5 nM PTX + 1 μM or 10 μM DEX for 24 hours were probed for MCP-1. HSP90 was used as loading control. Representative images of three independent experiments are shown. (**C**). Normalized MCP-1 expression. Bars indicate mean of three independent experiments ± SEM. Student’s *t* test was performed between each treatment and vehicle control. ***P = 0.016, ****P < 0.001. (**D**). Normalized MCP-1 expression. Bars indicate mean of three independent experiments ± SEM. Student’s t test was performed between pairs of treatments.***P = 0.004, **** P < 0.001, ^####^P < 0.001. (**E**). MCP-1 ELISA. ECs were grown in 6 well plates and treated with vehicle, 5 nM PTX or 5 nM PTX + 1 μM or 10 μM DEX for 24 hours. Secreted MCP-1 levels (pg/mL) in treated cell media were measured with an ELISA assay. Student’s t test was performed between pairs of treatments. *P < 0.001. ***P < 0.001, ##*P* < 0.001. (**F**). MCP-1 ELISA. ECs were grown in 6 well plates and treated with vehicle, 50 nM PTX or 50 nM PTX + 1 μM or 10 μM DEX for 24 hours. Secreted MCP-1 levels (pg/mL) in treated cell media were measured with an ELISA assay. Student’s t test was performed between pairs of treatments. **** P <0.001, **P = 0.004, ***P < 0.001. (**G**). Heatmap of cytokines released by ECs. Media was collected from HUVECs treated with vehicle (CTL) or with 5 nM or 50 nM PTX in combination with 0, 1, or 10 μM DEX. Cytokine levels (pg/ml) were measured with an ELISA assay. The heatmap is shaded such that blue and red indicate values that are at least two standard deviations below or above, respectively, the mean (white) of each row.

The RNAseq analysis showed that the transcription of MCP-1 (*CCL2*) is upregulated by up to 2-fold in ECs by PTX in a dose-dependent manner. Accordingly, treatment with 5 nM PTX doubled the level of MCP-1 protein in ECs (Fig. 3A–3D). Furthermore, co-treatment of DEX suppressed this PTX-mediated upregulation of MCP-1 in the EC lysates. MCP-1 is a secreted protein and was measured in the media of ECs using multiplex cytokine analysis. Conditioned media obtained from ECs pre-treated with 5 nM or 50 nM PTX showed a significant increase in MCP-1 levels compared to vehicle-treated EC (Fig. 3E–3G). Interestingly, PTX treatment upregulated a host of pro-inflammatory cytokines in the media of ECs, including IFN-α and IL-6, which were downregulated by DEX in a dose-dependent manner. Collectively, these results validated transcriptional perturbations induced by PTX in ECs and supported further *in vivo* examination of DEX.

### Peri-procedural treatment with DEX suppresses PTX-induced increase in MCP-1 levels

**Figure 4:**
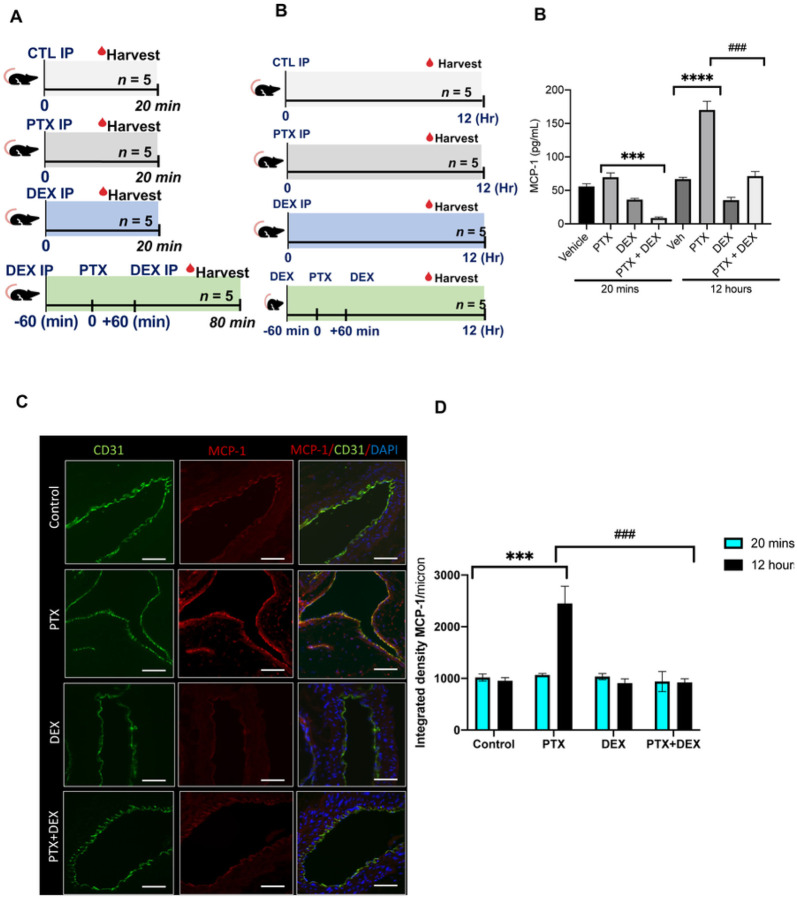
PTX induces upregulation of MCP-1 in a mouse model. (**A**). Overview of mouse model. A group of 8- to 12-week-old C57BL/6 female mice were given intraperitoneal (IP) injections of 5% DMSO control (CTL), 5 mg/kg PTX, 5 mg/kg DEX, or 5 mg/kg PTX + 5 mg/kg DEX (n = 10 per group). Mice co-administered PTX and DEX were given 5 mg/kg DEX both 60 minutes prior to and following injection of 5 mg/kg PTX. Five mice in each group were harvested at 20 minutes following the last injection (left panel) and the remainder at 12 hours following the last injection (right panel). (**B**). Serum MCP-1 levels (pg/ml) measured in mice given IP injection of DMSO control, PTX, DEX, or PTX+DEX and harvested at 20 minutes or 12 hours post-injection. Student’s t test was performed between pairs of treatments. *** *P* < 0.001, **** *P* < 0.001, ### *P* < 0.001. (**C**). Representative images of mice aorta harvested 12 hours post-injection stained for EC marker CD31 in green and MCP-1 in red. Four to five images were randomly taken (*n* = 5 mice/group). Scale bar: 100 μm. Original magnification 40X. (**D**). Integrated densities of normalized MCP-1 from images in Figure 4C and Supplemental Figure 2. Student’s *t* test was performed between pairs of treatments. ***P < 0.001, ^###^*P* < 0.001.

A group of C57BL/6 mice were randomized into four groups and administered 5 mg/kg PTX, 5 mg/kg DEX, or PTX + DEX intraperitoneally (IP). DMSO (vehicle) treated mice served as controls. Animals were harvested at either 20 minutes or 12 hours (Fig. 4A) to examine the immediate and delayed effects of PTX exposure. Serum levels of PTX were high in animals treated with PTX (average ± SEM of 6.8 ± 1.93 mmol/L) or PTX + DEX (4.99 ± 1.08 mmol/L). There was no significant difference in the PTX levels between these groups, and PTX was undetectable after 12 hours. Within 20 minutes of PTX injection, no significant increase in MCP-1 was detected; however, by 12 hours, PTX-injected mice showed a 3-fold increase in the levels of MCP-1 (P < 0.001) compared to vehicle-treated mice. This effect was entirely abrogated in mice treated with PTX + DEX (P < 0.001) (P < 0.001) (Fig. 4B).

**Figure 5:**
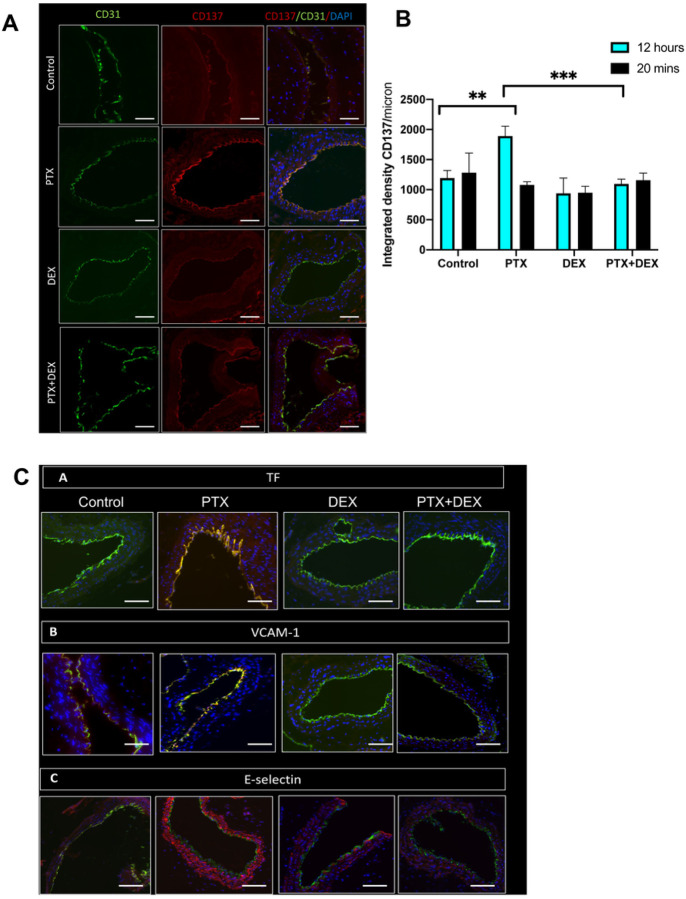
PTX induces expression of pro-inflammatory and pro-thrombotic proteins in mouse aortic ECs. (**A**). Representative images of mice aorta harvested 12 hours post-injection, stained for EC marker CD31 in green and CD137 in red. Four to five images were randomly taken (*n* = 5 mice/group). Scale bar: 100 μm. Original magnification 40X. (**B**). Integrated densities of normalized CD137 from images in Figure 5A and Supplemental Figure 3. Student’s t test was performed between pairs of treatments. **P = 0.018, ***P < 0.001. (**C**). Representative images of mice aorta harvested at 12 hours post-injection, stained for EC marker CD31 in green and TF, VCAM-1, or E-selectin in red. Four to five images were randomly taken (*n* = 5 mice/group). Scale bar: 100 μm. Original magnification 40X. (**D**). Integrated densities of normalized TF from images in Figure 5C and Supplemental Figure 4. Student’s t test was performed between pairs of treatments. ***P < 0.001, ****P < 0.001. (**E**). Integrated densities of normalized VCAM-1 from images in Figure 5C and Supplemental Figure 5. Student’s t test was performed between pairs of treatments. ***P < 0.001, ^###^*P* = 0.003. (**F**). Integrated densities of normalized E-selectin from images in Figure 5C and Supplemental Figure 6. Student’s t test was performed between pairs of treatments. ***P = 0.002, ****P <0.001.

We next examined whether PTX treatment altered MCP-1 levels in the aortic ECs of mice. The aorta of mice from different groups were stained and whole slide imaging was subjected to ImageJ analysis to quantitate the expression of protein using an integrated density (a composite of image intensity and the number of pixels normalized to the area). CD31 was used as an EC marker. There were no significant changes in MCP-1 expression at 20 minutes (**Supplementary Fig. 2**). However, at 12 hours, PTX-exposed mice showed a 2.3-fold (*P* < 0.001) increase in MCP-1 expression compared to control mice (Fig. 4C–4D), which was completely suppressed in the mice co-treated with PTX + DEX (*P* < 0.001). Similarly, no significant changes in CD137 levels were noted within 20 minutes of PTX treatment (**Supplementary Fig. 3**), but at 12 hours, a significant upregulation of CD137 was noted in the aortic ECs of PTX-treated mice (P = 0.018), which was absent in mice injected with PTX + DEX (P < 0.001) (Fig. 5A and 5B).

### DEX suppresses the induction of pro-thrombotic mediators by PTX

Pro-inflammatory mediators such as MCP-1 and CD137 induce atherothrombotic factors through downstream mediators such as tissue factor (TF), E-selectin and VCAM-1, converting normal vascular endothelium from an anti-coagulant to a pro-coagulant state^9,10^. TF is the primary trigger of the extrinsic coagulation cascade, and its upregulation increases the risk of plaque rupture and cardiovascular events. We therefore evaluated whether PTX increased the expression of these downstream pro-thrombotic mediators in ECs (Fig. 5C).

**Figure 6:**
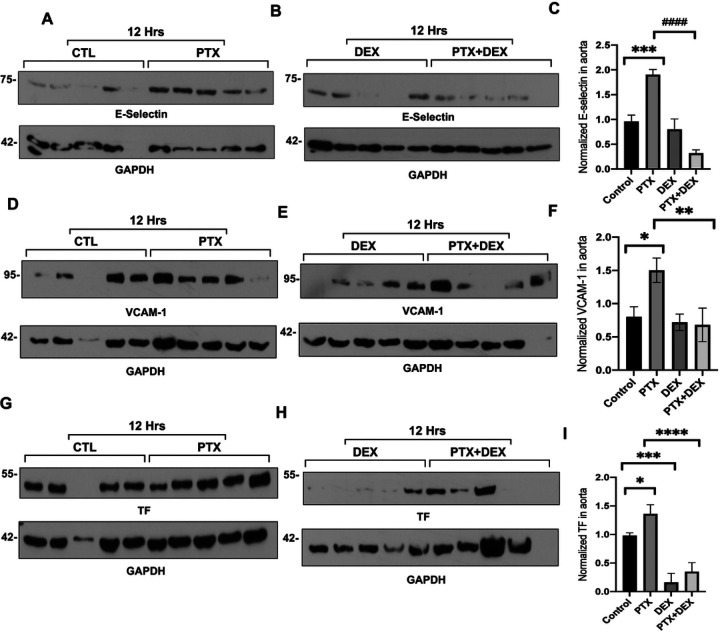
Mouse aortic lysates demonstrate increased prothrombotic protein expression after exposure to PTX. (**A**). Immunoblotting of E-selectin in mouse aorta. Mice were given IP injections of vehicle or 5 mg/kg PTX, aortas were harvested after 12 hours, and lysates were probed for E-selectin. GAPDH was used as a loading control. (**B**). Immunoblotting of E-selectin in mouse aorta. Mice were given IP injections of 5 mg/kg DEX or 5 mg/kg PTX + 5 mg/kg DEX, aortas were harvested after 12 hours, and lysates were probed for E-selectin. GAPDH was used as a loading control. (**C**). Normalized E-selectin expression. Bars indicate mean ± SEM. Student’s t-test was performed between pairs of treatments. ***P = 0.002, ^####^*P* < 0.001. (**D**). Immunoblotting of VCAM-1 in mouse aorta. Mice were given IP injections of vehicle or 5 mg/kg PTX, aortas were harvested after 12 hours, and lysates were probed for VCAM-1. GAPDH was used as a loading control. (**E**). Immunoblotting of VCAM-1 in mouse aorta. Mice were given IP injections of 5 mg/kg DEX or 5 mg/kg PTX + 5 mg/kg DEX, aortas were harvested after 12 hours, and lysates were probed for VCAM-1. GAPDH was used as a loading control. (**F**). Normalized VCAM-1 expression. Bars indicate mean ± SEM. Student’s t test was performed between pairs of treatments. *P = 0.044, PTX+DEX **P = 0.006. (**G**). Immunoblotting of TF in mouse aorta. Mice were given IP injections of vehicle or 5 mg/kg PTX, aortas were harvested after 12 hours, and lysates were probed for TF. GAPDH was used as loading control.

At 20 minutes after PTX injection, there was no significant change in TF levels between the PTX and control groups (**Supplementary Fig. 4**). However, by 12 hours, TF expression was significantly increased in the PTX group compared to control (P < 0.001), and this increase was abrogated by co-administration of PTX + DEX (P < 0.001) (Fig. 5C and 5D). A similar pattern was observed with respect to VCAM-1 and E-selectin expression (Fig. 5C, 5E and 5F). Immunoblotting was also performed using aortic lysates (n = 5 mice per group), with GAPDH serving as loading control (Fig. 6; **Supplemental Fig. 7**). At 20 minutes following PTX treatment, there were no alterations in the expression of TF, VCAM-1 and E-selectin. However, in aortic lysates obtained 12 hours after PTX treatment, the expression of these proteins was upregulated (~ 40–50%) compared to the control mice and these upregulations were suppressed in the PTX + DEX-treated group (Fig. 6A–6I).

## 4. Discussion

While the local anti-proliferative effects of PTX at the site of application in terms of both vascular smooth muscle cell proliferation and neointimal hyperplasia at the site of application are well understood^4^, the long-term consequences of PTX in the systemic circulation remain poorly understood. Our study shows that a single systemic exposure of PTX in mice is sufficient to upregulate pro-inflammatory proteins and atherothrombotic mediators, which are likely to trigger subsequent events leading to vascular toxicity.

Our in vivo experimental strategy probed two timepoints − 20 mins and 12 hours post-injection - to evaluate the immediate and delayed effects of PTX exposure. Within 20 mins after PTX injection, both the PTX and PTX-DEX-treated groups achieved comparable PTX blood levels (4.99–6.88 mmoles/L) which are similar to concentrations noted in previous studies^11,12^. Considering we identified the IC_50_ of PTX as 50 nM for ECs in-vitro, this systemic concentration implies that ECs in vivo were subjected to toxic drug levels.

MCP-1 or CCL2 is one of the key chemokines of the CC family, which regulates migration and recruitment of monocytes and increases cytokine production, adhesion molecule expression, and induction of reactive oxygen species (ROS) release in monocytes and ECs^13^. MCP-1 is expressed by a variety of activated cells (e.g., ECs, monocytes, and smooth muscle cells)^13^. While our work focused on ECs as a source of MCP-1 in blood, it does not rule out the possibility of an increase in MCP-1 from other cell types. Using genetic models, several studies have demonstrated the importance of MCP-1 in the development of atheromatous lesions and its consequences. For example, higher MCP-1 levels were detected in atherosclerotic lesions compared to normal human arteries^14,15^. Rupture of an atherosclerotic plaque is known to be associated with thrombotic events with potentially life-threatening complications. Studies have also demonstrated the proangiogenic effects of MCP-1 in the atherosclerotic plaque, which is known to increase plaque vulnerability^16^. MCP-1 induces matrix metalloproteinases leading to thinning of the atherosclerotic cap and increasing the risk of plaque rupture^17^. In addition, MCP-1 upregulates tissue factor (TF) synthesis, further contributing to thrombosis on the ruptured plaque and increasing the risk of potentially fatal coronary events^9^. Considering these known effects of MCP-1, the current data raise a possibility to relate the perceived long-term effect due to high levels of MCP-1 following PTX exposure, and this may likely contribute to the initiation of atherothrombotic processes.

Upregulation of CD137 in ECs leads to endothelial dysfunction, which subsequently increases the expression of adhesion molecules on ECs and pro-inflammatory cytokine production by them; both of these processes augment the recruitment and migration of leukocytes to exacerbate atherosclerotic process^7,8^. Based on the above findings, it is conceivable that a high-dose systemic exposure of PTX upregulates mediators that are known to trigger cascades of secondary events increasing the atherothrombotic process in the vasculature.

DEX is a synthetic glucocorticoid that exerts profound anti-inflammatory effects via different mechanisms including PDGF and IL-1β inhibition^18^. In addition to suppressing cytokine and interleukin signaling, DEX is known to reduce vSMC migration and proliferation and to downregulate adhesion molecule expression on ECs at the site of vessel injury^18^, all of which constitute the rationale of using DEX in this study. Our selection of DEX was further motivated by its ease of administration in the clinical setup before and after endovascular interventions.

Our study has a few limitations. We used cell culture and animal models to demonstrate the anti-inflammatory effect of DEX after PTX exposure. PTX dosing in our experiments was based on the overall drug content on a prototypic 200 mm length DCB approved for human use (Ranger^™^, Boston Scientific). Future studies can explore varied levels of PTX dosing followed by evaluation of systemic effects. Furthermore, detailed human studies are needed to assess the blood concentration of PTX during the deployment of PTX devices, which can inform animal experiments. Studies are also needed to rigorously evaluate the effect of PTX over a protracted period in higher models such as swine and the ones with comorbidities commonly seen in patients with high cardiovascular disease burden (such as obesity, diabetes etc.). Large animal studies are also needed to evaluate systemic toxicity when drug release is simulated following DCB angioplasty and accounting for biophysical and biochemical interactions.

In conclusion, this work demonstrates potential mediators of PTX-induced systemic toxicity emanating from a high-dose exposure, which emulates the spike of PTX observed with DCB angioplasty. This spike likely contributes to systemic toxicity, thereby enhancing the risk of atherothrombotic progression. The strategy to minimize PTX-induced toxic effects by systemically administering widely used anti-inflammatory compounds such as DEX should be explored further using other pre-clinical models and clinical studies. We also hope that this study paves the way for further analysis to broadly understand the mechanisms and mediators of PTX-induced adverse cardiovascular events observed in humans and the means to prevent them.

## Data Availability

The RNA sequencing dataset generated and analyzed during the current study is available in the Gene Expression Omnibus (GEO) repository, Series accession number GSE217550 (https://www.ncbi.nlm.nih.gov/geo/query/acc.cgi?acc=GSE217550).
